# “*Navigating the network”*: localising the lesion with the advent of lesion network mapping

**DOI:** 10.1007/s00415-023-12059-5

**Published:** 2023-10-26

**Authors:** Jack Sheppard, Ray Wynford-Thomas, Neil P. Robertson

**Affiliations:** 1 Department of Neurology, University Hospital of Wales, Heath Park, Cardiff, CF14 4XN UK; 2 Division of Psychological Medicine and Clinical Neuroscience, Department of Neurology, Cardiff University, University Hospital of Wales, Heath Park, Cardiff, CF14 4XN UK

“*Localising the lesion*” is a concept well-trodden by medical students beginning their journey in neurology and is considered an important step in developing a differential diagnosis. However, as demonstrated by the first two papers discussed this month, specific symptoms may be attributable to more than one lesion location. Furthermore, lesions occupying the same anatomical location do not always result in the same symptoms, as illustrated by paper three. Lesion network mapping, the theme linking all three papers, allows us to identify common neural pathways shared when heterogeneous lesions produce the same symptom. This may not only develop our understanding of neurophysiology and neuropathology in vivo but also provide more refined and validated neural targets for neurostimulation therapy. Whilst discussing each of the papers in this month’s journal club, we encourage you to consider whether, for assessment of some neurological diseases, we should move away from “*localising the lesion*” and towards “*navigating the network*”. This approach may facilitate a better appreciation of the brain as a series of upregulating and downregulating subcortical systems under cortical influence, that together form a carefully balanced network. As a result, when these networks are affected by pathology, symptoms often occur as the sum consequence of disruption rather than lesion location. With this may come an improved understanding of the use of deep brain stimulation (DBS), why it works, and how its application can be optimised.

## Mapping lesion-related epilepsy to a human brain network

Focal epilepsy is commonly caused by the development of brain lesions, such as those caused by stroke. However, not all strokes cause epilepsy, and though it remains unclear why, recent improvements in lesion mapping provide investigative opportunities. Schaper et al.’s retrospective multicentre case–control study compared 76 patients with post-stroke epilepsy (ischaemic) against two datasets of patients with ischaemic stroke not associated with epilepsy (*n* = 135 and *n* = 490). The primary outcome measure was the presence of epilepsy, with the objective of assessing whether lesion locations associated with epilepsy map to specific brain areas and/or networks.

Controlling for volume, lesion location mapping found no significant difference in post-stroke epilepsy incidence across lobes or vascular territories. More negative functional connectivity between lesion location and the basal ganglia and cerebellum was strongly associated with post-stroke epilepsy. Functional connectivity between the stroke lesion and the lesion network nodes (LNNs) from validation datasets [individuals with epilepsy secondary to non-stroke pathology (*n* = 772, 35% with epilepsy)] was significantly associated with post-stroke epilepsy, and this was consistent across all lesion types. There was also a significant difference across all pathologies in the proportion of patients with epilepsy throughout the three levels of lesion connectivity (high, moderate and low). To assess therapeutic importance, functional connectivity using a volume of tissue activated in 30 patients receiving anterior thalamic DBS was retrospectively used to compute the functional connectivity of the DBS sites and was compared to the LNNs generated by the study. Functional connectivity of participants’ DBS sites to the LNNs correlated with significantly reduced seizure frequency and remained significant after controlling for stimulation amplitude and volume. Reduction in seizure frequency was associated with increased functional connectivity with voxels in the LNNs.

**Comment**: Although lesion location mapping did not identify anatomical areas most at risk for developing epilepsy, it did suggest that lesions associated with epilepsy are part of a specific neural network. Negative functional connectivity between these areas and regions in the basal ganglia and cerebellum is independently associated with epilepsy. This supports animal studies suggesting epilepsy is a neurological network disorder and that this network is common across multiple aetiologies of focal epilepsy, thus suggesting why DBS is successful across such aetiologies. However, all datasets were retrospective and so variables such as stroke severity, seizure frequency and predisposing factors could not be controlled for. Furthermore, the current approach to lesion network mapping uses connectome data from healthy participants which may not accurately represent pathological brains. Lastly, though this study provides insight into a variety of types of focal epilepsy, the generalisability of the results is unclear. Prospective studies assessing a variety of epilepsy aetiologies (ideally considering a connectome created from averaging neural pathways from pathological brains) are needed before utilising network mapping for treatment or prognostication.

Schaper FM et al. (2023) JAMA Neurol. 80(9):891–902

## A neural network for tics: insights from causal brain lesions and deep brain stimulation

Tics are repetitive sudden movements or sounds, visually similar to voluntary actions but acontextual to environment. Though cortical and subcortical areas are associated with tics, their causal role remains unclear. The aim of this combined methods literature review and multicentre retrospective case–control study was to describe neural networks linked to tic generation using lesion and DBS network mapping. The first primary outcome was the presence of a shared neural network common across most sample brain scans of individuals with tics. The second was the review of the network’s therapeutic relevance by assessing the association between the clinical outcome in a retrospective thirty-patient cohort with Tourette’s from three European centres with the proximity of DBS sites to the identified network.

22 cases of new-onset lesion-attributable tics were identified through a literature review using predefined inclusion criteria. Lesion networks were mapped to define regions most connected to the majority of tic-inducing lesions (19/22). Comparing this with those generated for distinct lesions from the Harvard Lesion Repository, conjunction analysis identified the associative-limbic functional zone of the anterior striatum within the anterior putamen (close to an area commonly targeted in DBS) as being both sensitive and specific to tics. DBS network maps created for each patient with Tourette’s were compared to both the sensitive and specific tic lesion network maps to assess how DBS connectivity strength might explain tic improvement. Connectivity strength between the lesion network and the DBS sites significantly correlated with tic improvement in both combined and separated thalamic and pallidal DBS cohorts. There was a significant correlation between the degree of connectivity of the DBS sites and the lesion network maps and clinical improvement. Mapped brain regions with positive clinical outcomes shared by both pallidal and thalamic DBS sites displayed a network that correlated with the network produced by lesion-mapping tic-inducing brain lesions.

**Comment**: The main limitations of this study are the retrospective and literature-based collection of patient cases (for which causality between lesion and tic development cannot be verified) and the retrospective collection of DBS cases. Another significant limitation is the use of a connectome generated by healthy volunteers to create the lesion and DBS network. This assumes that pathological brains have the same functional network as in health and limits patient-specific application of results. Validation of the lesion network map is limited by the fact it was performed retrospectively on a cohort (those with Tourette’s who may or may not have an associated lesion) not defined as the original target population (patients with tics secondary to lesion). Although this study suggests a template of effective target areas for DBS for tic management, prospective validation in multiple populations is required to validate the role of the identified lesion network in the development of tics and the relevance of DBS.

Ganos C et al. (2022) Brain 145(12):4385–4397

## Pathological laughter and crying: insights from lesion network-symptom-mapping

Pathological laughing and crying (PLC) is characterised by short, intense and frequent bursts of uncontrollable laughing or crying provoked by incongruous stimuli or maligned with mood, and can occur due to central nervous system pathologies such as stroke, tumour or neurodegenerative conditions. The aim of this retrospective case–control study was to establish the nature of the neural network involved in PLC by mapping heterogeneous lesion locations and then interrogating this network using case studies to examine whether there is an association between it and the theorised emotional/volitional systems.

Following a literature review for PLC attributable to focal intra-axial lesions delineable to a lesion shown on imaging, 70 cases were included. A further literature review revealed 46 cases of volitional facial paresis (VFP) or Foix-Chavany-Marie (FCM) and 15 cases of emotional facial paresis (EFP). 270 control cases were taken retrospectively from two prospective cohorts recruited to study post-stroke depression. Lesion masks for those with PLC (2D scans) were manually delineated on a standard brain. 100 healthy controls from the *human connectome* project were used to identify potentially affected brain networks and lesion network symptom mapping (LNSM) was performed.

LNSM showed a significant difference between the lesion networks of the 70 cases with PLC and the 270 controls. Those areas with positive lesion-dependent connectivity (the ‘positive PLC subnetwork’) included the brainstem, hypothalamus, striatum, cerebellum, cingulate and temporal cortices. Areas with negative lesion-dependent connectivity (the ‘negative PLC subnetwork’) included the sensorimotor cortices, parietal and occipital regions and cerebellum, but not the basal ganglia or brainstem. 14/15 lesions causing EFP mapped onto the ‘positive PLC subnetwork’ and 41/46 of those causing VFP mapped onto the ‘negative PLC subnetwork’ (statistically significant). From this, the authors hypothesise the existence of a two-hit model whereby, secondary to a lesion, the negative PLC subnetwork (volitional system) fails to exert its normal inhibitory cortical control over the positive PLC subnetwork (emotional system) which, due to the pathology, is itself dysfunctional, and PLC results. This dual system dysfunction may arise due to a combination of direct lesion effect and/or diaschisis depending on the lesion’s location and size.

**Comment**: This is the first study to show that different lesion locations influence a common neural network in the generation of PLC, but has some limitations. These again include the use of the *human connectome*, thereby assuming neural networks are similar in healthy individuals and those with pathology, and the use of retrospectively collected cases. Furthermore, the authors state the prospectively recruited control patients with stroke were not assessed for PLC, resulting in false negatives. Finally, the use of hand-drawn lesions in 2D slices might have limited lesion localisation accuracy in the brainstem in particular, but the exclusion of pontine lesions left the generated PLC network unchanged.

Klingbeil J et al. (2021) Brain 144(10):3264–3276

**Summary:** Given the common limitations of the three papers discussed this month, prospective case–control studies using a pathological human brain connectome, themselves validated prospectively, are required to better understand the full relevance of the conclusions drawn. Nonetheless, it seems clear that symptom manifestation in some neurological conditions is not solely attributable to lesion location but significantly influenced by the role of neural networks. In such conditions, we propose integrating or prioritising a “*navigating the network*” approach alongside or over the established current standard of clinical reasoning in neurology: “*localising the lesion*”. This may improve not only the way we impart our knowledge of the nervous system to our medical students but also our clinical diagnostic process, understanding, prognostication and management of those conditions whereby neural networks are shown to be especially relevant, with particular respect to the optimal therapeutic application of DBS. At the very least, these papers serve to illustrate the continued value of lesion network mapping as a research tool, and might even offer insight into potential future clinical use for the individualised application of DBS as personalised medicine continues to be refined.

